# Supplementary material for: Despite vaccination, China needs non-pharmaceutical interventions to prevent widespread outbreaks of COVID-19 in 2021
Source: Nat Hum Behav. 2021 Jun 22;5(8):1009–20. doi: 10.1038/s41562-021-01155-z (PMC8373613; doi:10.1038/s41562-021-01155-z)
Supplement: Supplementary file 1 — Supplementary Files 1–7, Supplementary Figs. 1–8 and Supplementary Tables 1–4. [file 41562_2021_1155_MOESM1_ESM.pdf]

nature portfolio

https://doi.org/10.1038/s41562-021-01155-z

Supplementary information

Despite vaccination, China needs non-pharmaceutical interventions to prevent widespread outbreaks of COVID-19 in 2021

In the format provided by the authors and unedited

Supplementary Information

Despite vaccination, China needs non-pharmaceutical interventions to prevent widespread outbreaks of COVID-19 in 2021

Juan Yang[1,2,8], Valentina Marziano[3,8], Xiaowei Deng[1], Giorgio Guzzetta[3], Juanjuan Zhang[1,2], Filippo Trentini[3], Jun Cai[1], Piero Poletti[3], Wen Zheng[1], Wei Wang[1], Qianhui Wu[1], Zeyao Zhao[1], Kaige Dong[1], Guangjie Zhong[1], Cécile Viboud[4], Stefano Merler[3,9], Marco Ajelli[5,6,9], Hongjie Yu[1,2,7,9]

[1]Shanghai Institute of Infectious Disease and Biosecurity, School of Public Health, Fudan University, Shanghai, China. [2]Fudan University, Key Laboratory of Public Health Safety, Ministry of Education, Shanghai, China. [3]Center for Health Emergencies, Bruno Kessler Foundation, Trento, Italy. [4]Division of International Epidemiology and Population Studies, Fogarty International Center, National Institutes of Health, Bethesda, MD, USA. [5]Department of Epidemiology and Biostatistics, Indiana University School of Public Health, Bloomington, IN, USA. [6]Laboratory for the Modeling of Biological and Socio-technical Systems, Northeastern University, Boston, MA, USA. [7]Department of infectious diseases, Huashan Hospital, Fudan University, Shanghai, China. [8]These authors contributed equally: Juan Yang, Valentina Marziano. [9]These authors jointly supervised this work: Stefano Merler, Marco Ajelli, Hongjie Yu. e-mail: marco.ajelli@gmail.com; yhj@fudan.edu.cn

Contents

Supplementary file 1. SARS-CoV-2 transmission and vaccination models 3
Supplementary file 2. Estimating of the scaling factor for transmissibility in the absence of NPIs (β) 9
Supplementary file 3. Priority population of COVID-19 vaccination 10
Supplementary file 4. Estimating the proportion of laboratory-confirmed COVID-19 symptomatic cases requiring hospitalization and death for individuals with and without underlying conditions 11
Supplementary file 5. Data analysis 14
Supplementary file 6. Additional Figures 15
Supplementary file 7. COVID-19 vaccine doses distributed over time in China 23
References 24

Supplementary file 1. SARS-CoV-2 transmission and vaccination models

We developed a model of SARS-CoV-2 transmission and vaccination, based on an age-structured stochastic susceptible-infectious-removed (SIR) scheme, accounting for heterogeneous mixing patterns by age as estimated in Shanghai [1]. The Chinese population was distributed in 18 age groups (17 5-year age groups from 0 to 84 years and one age group for individuals aged 85 years or older) [2]. Each age group was further split into two subgroups: individuals with or without underlying conditions, where the former was considered to be associated with an increased risk of severe outcome of COVID-19 [3].

In the main analysis, susceptibility to SARS-CoV-2 infection was assumed to be heterogeneous across ages. Children under 15 years of age were considered less susceptible to infection compared to adults aged 15 to 64 years, while the elderly more susceptible [4]. Asymptomatic and symptomatic individuals were assumed to be equally infectious [4,5], and infectiousness was also assumed to be the same across age groups [4,5].

Vaccine is administered with a two-dose schedule. In the baseline model, we assumed that: i) vaccination reduces susceptibility to SARS-CoV-2 infection; ii) only susceptible individuals are eligible for vaccination, i.e., we excluded all individuals that have experienced SARS-CoV-2 infection; iii) duration of vaccine-induced protection lasts longer than the time horizon considered (2 years).

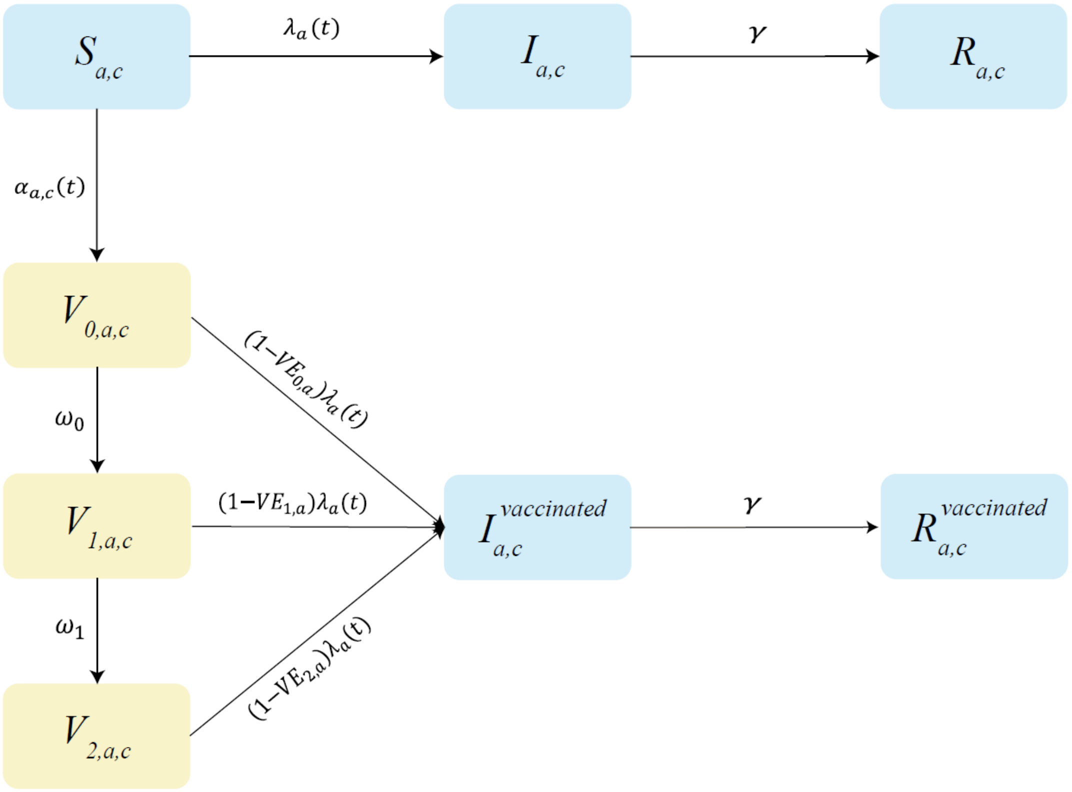

Supplementary Figure. 1 Schematic representation of the baseline model. Blue, and yellow rectangles describe the SARS-CoV-2 transmission model, and vaccination model, respectively. Transitions occur within each population class defined by a and c, where a represents the age group and c identifies the absence/presence of underlying conditions, the latter associated with higher risk of sever outcomes of SARS-CoV-2 infections. S denotes susceptible individuals; I infected individuals; R recovered/removed individuals. Parameters of the transmission model include: the time- and age-dependent force of infection $\lambda_a(t)$ and the recovery rate from infection (γ). V_0 denotes individuals vaccinated with the 1st dose (never experienced infection with SARS-CoV-2); V_1 denotes vaccinated with the 2nd dose (no protection yet); V_2 denotes vaccinated with the 2nd dose (protected). Parameters of the vaccination model include: the time-, age- and group-dependent probability of being vaccinated ($\alpha_{a,c}(t)$); the interval between administration of the first and second dose ($1/\omega_0$); the delay of ramp-up of the 2nd dose ($1/\omega_1$); the age-dependent vaccine efficacy after the 1st dose of vaccination ($VE_{0,a}$), which is assumed to be 0; the age-dependent efficacy right after administration of the 2nd dose ($VE_{1,a}$), which is assumed to be 0; the vaccine efficacy after ramp-up of the 2nd dose ($VE_{2,a}$).

The baseline model is schematically represented in Supplementary Figure 1 and it is described by the following differential systems:

$$\begin{cases} S'_{a,c}(\mathrm{t}) = -\lambda_a(t)S_{a,c}(t) - \alpha_{a,c}(t)S_{a,c}(t) \\ I'_{a,c}(\mathrm{t}) = \lambda_a(t)S_{a,c}(t) - \gamma I_{a,c}(t) \\ R'_{a,c}(\mathrm{t}) = \gamma I_{a,c}(t) \\ V'_{0,a,c}(\mathrm{t}) = \alpha_{a,c}(t)S_{a,c}(t) - \left(1-VE_{0,a}\right)\lambda_a(t)V_{0,a,c} - \omega_0 V_{0,a,c}(t) \\ V'_{1,a,c}(\mathrm{t}) = \omega_0 V_{0,a,c}(t) - \left(1-VE_{1,a}\right)\lambda_a(t)V_{1,a,c} - \omega_1 V_{1,a,c}(t) \\ V'_{2,a,c}(\mathrm{t}) = \omega_1 V_{1,a,c}(t) - \left(1-VE_{2,a}\right)\lambda_a(t)V_{2,a,c} \\ I_{a,c}^{\mathrm{vaccinated}\,\prime}(\mathrm{t}) = \lambda_a(t)[\left(1-VE_{0,a}\right)V_{0,a,c} + \left(1-VE_{1,a}\right)V_{1,a,c} + \left(1-VE_{2,a}\right)V_{2,a,c}] - \gamma I_{a,c}^{\mathrm{vaccinated}}(t) \\ R_{a,c}^{\mathrm{vaccinated}\,\prime}(\mathrm{t}) = \gamma I_{a,c}^{\mathrm{vaccinated}}(t) \end{cases}$$

where:

- $S_{a,c}$ represents the number of susceptible to SARS-CoV-2 infection in the population class *{a,c}*, where a represents the age group and c identifies the absence/presence of underlying conditions.
- $I_{a,c}$ represents the number of infectious unvaccinated individuals in the population class *{a,c}*.
- $R_{a,c}$ represents the number of unvaccinated individuals in the population class *{a,c}* who recovered from infection.
- $V_{0,a,c}, V_{1,a,c}, and\ V_{2,a,c}$ represent the number of vaccinated individuals in each ramp-up stage. In particular,
  1) $V_{0,a,c}$ denotes individuals in the population class *{a,c}* vaccinated with the first dose. In the main analysis, we assumed that the second dose is administered 21 days after the 1st dose. So $1/\omega_0 = 21$ days.
  2) $V_{1,a,c}$ denotes individuals in the population class *{a,c}* vaccinated with the second dose, for whom the 2nd dose is not effective yet. We assumed that the second dose becomes effective 14 days after administration, so $1/\omega_1 = 14$ days.
  3) $V_{2,a,c}$ denotes individuals in the population class *{a,c}* vaccinated with the second dose for whom vaccination is effective.
- $I_{a,c}^{\mathrm{vaccinated}}$ represents the number of infectious individuals in the population class *{a,c}* among those who have already received at least one dose of vaccination.
- $R_{a,c}^{\mathrm{vaccinated}}$ represents the number of individuals in the population class *{a,c}* who developed infection despite having received vaccination (one or more doses).

Susceptible individuals are exposed to a time and age-dependent force of

infection $\lambda_a(t)$ which is defined as:

$$\lambda_a(t) = (1-\varphi)\beta r_a \sum_{\tilde{a}} C_{a,\tilde{a}} \frac{\sum_c [I_{\tilde{a},c}(t) + I_{\tilde{a},c}^{vaccinated}(t)]}{\sum_c N_{\tilde{a},c}}$$

where:

- β is a scaling factor shaping SARS-CoV-2 transmissibility in the absence of non-pharmaceutical interventions (no NPIs, Effective reproductive number R_t =2.5), such as social distancing, school closure, and case isolation.
- φ is a coefficient representing the reduction in transmissibility due to NPIs.
- r_a is the relative susceptibility to SARS-CoV-2 infection at age a: $r_a = 0.58$ (95%CI 0.34-0.98) when $a < 15$; $r_a = 1$ for $15 \leq a < 65$; $r_a = 1.65$ (95%CI 1.03-2.65) when $a \geq 65$ [4,5].
- $C_{a,\tilde{a}}$ represents the age-group-specific contact matrix, whose entries describe the mean numbers of persons in age group $\tilde{a}$ encountered by an individual of age group a per day.
- $N_{\tilde{a},c}$ represents the number of individuals in the population class *{ã,c}*.

For all infectious compartments, the average duration of infectiousness $(1/\gamma)$ is set equal to the average generation time (5.5 days)[4].

At each time *t*, the first dose of vaccination is administered to a fraction $\alpha_{a,c}(t)$ of susceptible individuals in the population class *{a,c}*:

$$\alpha_{a,c}(t) = \frac{d_{a,c}(t)}{S_{a,c}(t)}$$

where $d_{a,c}(t)$ represents the number of (first) vaccine doses to be administered to individuals of the population class *{a,c}* at time *t* under the considered vaccination scenario.

The daily number of first doses $d_{a,c}(t)$ to be administered to the population class *{a,c}* is computed by taking into account: i) the assumed priority order; ii) the assumed vaccination coverage, i.e. the fraction of population that is expected to be vaccinated at the end of the program; ii) the constraints on the daily vaccination capacity. In particular, we assume that half of the daily capacity is allocated to first doses, i.e.:

$$\sum_{a,c} d_{a,c}(t) = \text{(daily vaccination capacity)}/2$$

and the remaining half to second doses.

Vaccinated individuals $V_{i,a,c}$ (i=0,1,2) can develop infection, but their susceptibility to infection is reduced by a factor $(1 - VE_{i,a})$, where $VE_{i,a}$ represents the age-specific vaccine efficacy associated to the *i*-th vaccination stage. In the main analysis,

the age-dependent vaccine efficacy after the 1st dose of vaccination ($VE_{0,a}$) was assumed to be 0; the age-dependent efficacy right after administration of the 2nd dose ($VE_{1,a}$) was also assumed to be 0; while the vaccine efficacy after ramp-up of the 2nd dose ($VE_{2,a}$) was assumed to be 80% for individuals aged 20-59 years and 40% for all other age groups. Simulation results discussed in the main text and in the following sections were obtained by using a stochastic version of the model described above. A summary of model parameters and data sources is presented in Supplementary Table 1.

Supplementary Table 1. Description of key parameters used in the model.

Description of parameter	Values in the baseline analysis	Sensitivity analyses (SA)
**Epidemiology**		
Generation time ($1/\gamma$)	5.5 days (95%CI 1.7, 11.6) [*4]	/
Relative susceptibility to infection at age a (r_a)	$r_a = 0.58$ (95%CI 0.34-0.98) when $a < 15$; $r_a = 1$ for $15 \le a < 65$; $r_a = 1.65$ (95%CI 1.03-2.65) when $a \ge 65$ [4]	Homogenous susceptibility ***(SA19)***
Age-group-specific contact matrix ($C_{a,\tilde{a}}$)	Contact matrix for Shanghai before pandemic [1]	Contact matrix for Shanghai at the last stage of the first wave of pandemic in Wuhan, China (March 2020) ***(SA13)***[6]
Effective reproductive number (R_t)	1.1, 1.3, 1.5, and 2.5 [7-12]	/
**Vaccination**		
Interval between the administration of 1st dose and 2nd dose ($1/\omega_0$)	21 days[* 13]	14 and 28 days ***(SA16-SA17)*** [13]
Delay between administration of the 2nd dose of vaccination and the achievement of the expected VE ($1/\omega_1$)	14 days[* 13]	/
Expected vaccine efficacy for adults aged 20-59 years ($VE_{2,a}$)	80% [14] for a vaccine with partial protections	60% ***(SA9)*** and 90% ***(SA10);*** and for an all-or-nothing vaccine ***(SA18)***
Expected vaccine efficacy reduction for <20 and ≥60 years	50% [13,15]	0%, indicating the same vaccine efficacy ***(SA24)***
Vaccination capacity (daily doses administered)	6 million (Assumed based on 2009 influenza pandemic vaccination) [16]	1.3 ***(SA20)***, 10 ***(SA21)***, 15 ***(SA22)*** and 30 million ***(SA23)***
Vaccine coverage	Homogenous across age groups: 70% [3]	Homogenous across age groups: 50% (***SA4***) or 90% ***(SA5)*** [3]*;* 70% for adults ⩾20

		years, and then 50% for others (***SA6***); 90% for adults ⩾20 years, and then 70% for others (***SA7***); 70% for adults ⩾20 years, and then no vaccination for others (***SA8***)
Duration of immunity ($1/\omega_2$)	Lifelong (i.e., the immunity lasts more than the time horizon considered: 730 days) (Assumed)	6 months ***(SA12)***, or 1 year ***(SA25)*** *
Delay between start of simulations and start of vaccination	15 days (Assumed)	SARS-CoV-2 infections leading to an outbreak are imported when 10% (***SA1***), 20% (***SA2***), and 30% (***SA3***) of the Chinese population has already been vaccinated.
**Disease burden**		
Proportion of infections that develop symptoms (π)	18.1%, 22.4%, 30.5%, 35.5%, and 64.6% separately for 0-19, 20-39, 40-59, 60-79, and 80+ years[17]	/
Proportion of laboratory-confirmed symptomatic cases requiring hospitalization (σ)	Overall: 40.0%, 29.2%, 33.3%, and 33.8% separately for 0-19, 20-39, 40-59, and 60+ years[18]; estimates for individuals with/without underlying conditions shown in Supplementary Information File 4	/
Proportion of hospitalized cases requiring ICU (ρ)	0, 2.2%, 7.2%, 20.9% separately for 0-14, 15-49, 50-64, and 65+ years[19]	/
Fatality ratio among laboratory-confirmed symptomatic cases (μ)	Overall: 0.51%, 0.65%, 2.38%, and 10.52% separately for 0-19, 20-39, 40-59, and 60+ years[18]; estimates for individuals with/without underlying conditions shown in Supplementary Information File 4	/

*mean value.

Supplementary file 2. Estimating of the scaling factor for transmissibility in the absence of NPIs (β)

The reproduction number can be computed as the dominant eigenvalue of the Next Generation Matrix (NGM) [20] associated with the dynamical system considered:

$$(NGM)_{a,\tilde{a}} = \frac{\beta}{\gamma} r_a C_{a,\tilde{a}}$$

We assumed a reproduction number in the absence of NPIs $R_t(\text{no NPIs}) = 2.5$. Given the value of $R_t(\text{no NPIs})$, the distribution of the age-specific susceptibility profile (r_a) and the distribution of the bootstrapped contact matrix, we computed the distribution of β analytically.

When considering a set of NPIs that are capable to bring the reproduction number to a value R_t(NPIs)< R_t(no NPIs), we used the distribution of β obtained in the absence on NPIs, rescaled by a factor $(1 - \varphi)$ where

$\varphi = 1 - R_t$(NPIs)/ $R_t(\text{no NPIs})$.

Supplementary file 3. Priority population of COVID-19 vaccination

Supplementary Table 2. Priority population of COVID-19 vaccination*

Tier of vaccination	Baseline (First prioritization to old adults and individuals with underlying conditions)	First prioritization to old adults (***SA26***[ǂ])	First prioritization to working-age groups (***SA27***)	First prioritization to school-age groups (***SA28***)
1	Healthcare workers (No=10.7 million)			
2	Law enforcement and security workers, personnel in nursing home and social welfare institutes, community workers, workers in energy, food and transportation sectors, etc. (No=36.8 million)			
3	Adults ≥ 60 years of age with underlying conditions, and adults ≥ 80 years of age without underlying conditions (No.=162.9 million)	Adults ≥ 60 years of age (No= 248.6 million)	Individuals aged 20-59 years (No= 807.2 million)	School-age children (No= 237.4 million)
4	Older adults aged 60-79 years without underlying conditions, individuals aged < 60 years with pre-existing medical conditions, and pregnant women (No.=401.2 million)	Individuals aged 20-59 years (No= 807.2 million)	School-age children (No= 237.4 million)	Individuals aged 20-59 years (No= 807.2 million)
5	Individuals aged 20-59 years without underlying conditions (No.=525.8 million)	School-age children (No= 237.4 million)	Adults ≥ 60 years of age (No= 248.6 million)	Adults ≥ 60 years of age (No= 248.6 million)
6	School-age children and younger children ≤5 years (No.=301.9 million)	Younger children≤5 years (No= 98.7 million)		

[*]Healthcare workers and the other essential workers listed here are fixed in Tier 1 and Tier 2 of vaccination, and thus would be vaccinated before other subgroups. [ǂ]Sensitivity analysis.

Supplementary file 4. Estimating the proportion of laboratory-confirmed COVID-19 symptomatic cases requiring hospitalization and death for individuals with and without underlying conditions

In order to quantify the different burden of COVID-19 in individuals with and without underlying conditions (such as chronic respiratory disease, heart disease, cardio-cerebrovascular disease, hypertension, diabetes, chronic renal diseases, chronic liver disease, cancer, and obesity [3]), we estimated the hospitalization and death rates for the two subgroups in China, using below data: 1) the overall age-specific hospitalization and death rates among symptomatic cases independent from the presence of underlying conditions in China [18]; 2) the proportion of symptomatic cases hospitalized/died in the two subgroups as obtained from the Lombardy region of Italy [17,21].

The age-specific proportions of laboratory-confirmed symptomatic cases requiring hospitalization for individuals with ($\sigma_{a,u}$) and without ($\sigma_{a,nu}$) underlying conditions were computed respectively as:

$$\sigma_{a,u} = s \cdot h_u^{ITA} \cdot \Delta_a$$

$$\sigma_{a,nu} = s \cdot h_{nu}^{ITA} \cdot \Delta_a$$

Where,

- h_u^{ITA} and h_{nu}^{ITA} separately denote the proportion of hospitalized among symptomatic cases with and without underlying conditions as estimated from Lombardy data (Supplementary Table 3) [17,21,22].
- $\Delta_\mathbf{a}$ denotes the age-specific proportion of laboratory-confirmed symptomatic cases requiring hospitalization as estimated for China independently from the presence of underlying conditions [18].
- the scale factor s is determined in such a way to minimize the root mean square error between Δ_a and $\widetilde{\Delta_a} = P_{a,u} \cdot \sigma_{a,u} + P_{a,nu} \cdot \sigma_{a,nu}$. $P_{a,u}$ and $P_{a,nu}$ denote the proportions of individuals of age with and without underlying conditions in China, respectively [3].

Analogously, the age-specific fatality ratios among laboratory-confirmed symptomatic cases for individuals with ($\mu_{a,u}$) and without ($\mu_{a,nu}$) underlying conditions are computed respectively as:

$$\mu_{a,u} = v \cdot m_u^{ITA} \cdot M_a$$

$$\mu_{a,nu} = v \cdot m_{nu}^{ITA} \cdot M_a$$

Where,

- m_u^{ITA} and m_{nu}^{ITA} denotes the proportion of cases with fatal outcomes among symptomatic cases with and without underlying conditions as estimated from Lombardy data (Supplementary Table 3) [17,21,22].
- M_a denotes the age-specific fatality ratio among laboratory-confirmed symptomatic cases as estimated for China independently from the presence of underlying conditions [18].
- the scale factor v is determined in such a way to minimize the root mean square error between M_a and $\widetilde{M_a} = P_{a,u} \cdot \mu_{a,u} + P_{a,nu} \cdot \mu_{a,nu}$.

Estimates were reported in Supplementary Table 4.

Supplementary Table 3. Proportion of laboratory-confirmed symptomatic cases requiring hospitalizations and having fatal outcomes among patients with or without underlying conditions*

	With underlying conditions	Without underlying conditions	Total
Laboratory-confirmed symptomatic cases	44446	44092	88538
Laboratory-confirmed symptomatic cases requiring hospitalizations	29593	17800	47393
Laboratory-confirmed symptomatic cases with fatal outcomes	13683	3095	16778
Proportion of hospitalization among laboratory-confirmed symptomatic cases (%)	h_u^{ITA} =66.6	h_{nu}^{ITA} =40.4	53.5
Proportion of laboratory-confirmed symptomatic cases with fatal outcomes (%)	m_u^{ITA} =30.8	m_{nu}^{ITA} =7	19

* The data were obtained from the line list of COVID-19 patients in the Lombardy region of Italy, with underlying diseases including chronic respiratory disease, cardiovascular disease, metabolic disease and cancer [17,21,22].

Supplementary Table 4. Estimated hospitalization and death rates for individuals with and without underlying conditions in China.

	With/without underlying conditions	With underlying conditions	Without underlying conditions
Proportion of laboratory-confirmed symptomatic cases requiring hospitalizations (%)	Δ_a [18]	$\sigma_{a,u}$	$\sigma_{a,nu}$

0-19 years	40	51.9	31.5
20-39 years	29.2	37.9	23.0
40-59 years	33.3	43.2	26.2
60+ years	33.8	43.8	26.6
Fatality ratio among laboratory-confirmed symptomatic cases (%)	M_a[18]	$\mu_{a,u}$	$\mu_{a,nu}$
0-19 years	0.51	0.66	0.15
20-39 years	0.65	0.84	0.19
40-59 years	2.38	3.06	0.70
60+ years	10.52	13.53	3.07

Supplementary file 5. Data analysis

For each scenario, 200 stochastic model realizations were performed. The outcome of these simulations determined the distributions of the number of symptomatic infections, hospitalizations, ICU admissions, and deaths. 95% confidence intervals were defined as quantiles 0.025 and 0.975 of the estimated distributions. We used a Bayesian approach to estimate R_t from the time series of symptomatic cases by date of symptom onset and the distribution of the serial interval. The methods have been described previously [23].

To estimate R_t, we assumed that the daily number of new cases (by date of symptom onset), including locally acquired infections L(t), can be approximated by a Poisson distribution according to the equation.

$$L(t) \sim Pois\left(R(t)\sum_{s=1}^{t}\varphi(s)C(t-s)\right)$$

Where,

- $C(t)$, with t from 0 to T, is the daily number of locally acquired new cases, by date of symptom onset;
- $R(t)$ is the net reproduction number at time t;
- $\varphi(s)$ is the distribution of the generation time (corresponding to the distribution of the serial interval) calculated at time s.

The likelihood $\mathscr{L}$ of the observed time series of cases from day 1 to T conditional on $C(0)$ is thus given by

$$\mathcal{L} = \prod_{t=1}^{T} P\left(L(t); R(t)\sum_{s=1}^{t}\varphi(s)C(t-s)\right)$$

where $P(k;\ \lambda)$ is the probability mass function of a Poisson distribution (i.e., the probability of observing k events if these events occur with rate λ).

We used Metropolis-Hastings MCMC sampling to estimate the posterior distribution of $R(t)$. The Markov chains were run for 1,000,000 iterations, assuming non-informative prior distributions of $R(t)$ (flat distribution in the range (0-1000]). Convergence was checked by visual inspection by running multiple chains starting from different starting points.

Supplementary file 6. Additional Figures

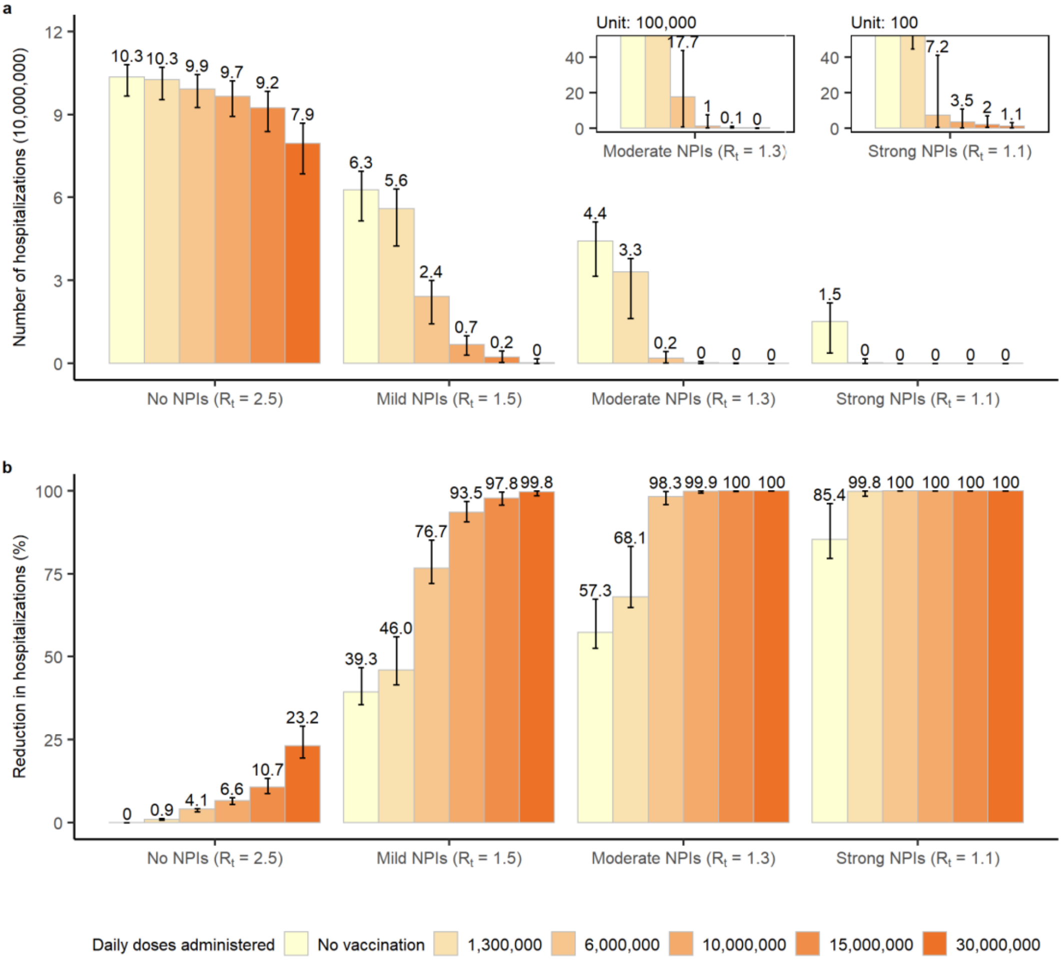

Supplementary Figure. 2 Impact of daily doses administered on COVID-19 hospitalizations.

a) Cumulative number of COVID-19 hospitalizations as estimated in the different scenarios under progressively increasing values of the daily vaccination capacity; b) Proportion of hospitalizations averted compared to the *reference scenario*, i.e., no vaccination + no NPIs with R_t=2.5 at the beginning of the outbreak. Number denotes median, and error bars denote quantiles 0.025 and 0.975.

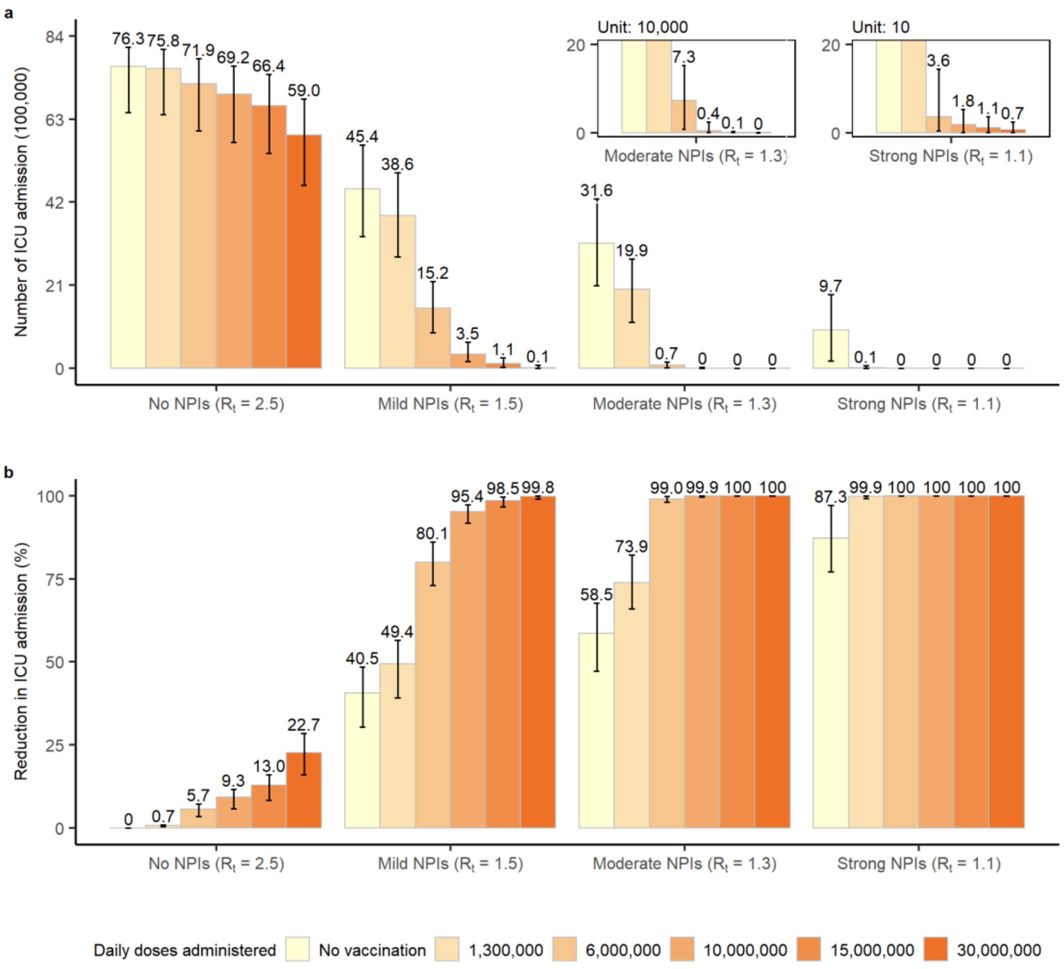

Supplementary Figure.3 Impact of daily doses administered on COVID-19 ICU admissions.

a) Cumulative number of COVID-19 ICU admissions as estimated in the different scenarios under progressively increasing values of the daily vaccination capacity; b) Proportion of ICU admissions averted compared to the *reference scenario*, i.e., no vaccination + no NPIs with R_t=2.5 at the beginning of the outbreak. Number denotes median, and error bars denote quantiles 0.025 and 0.975.

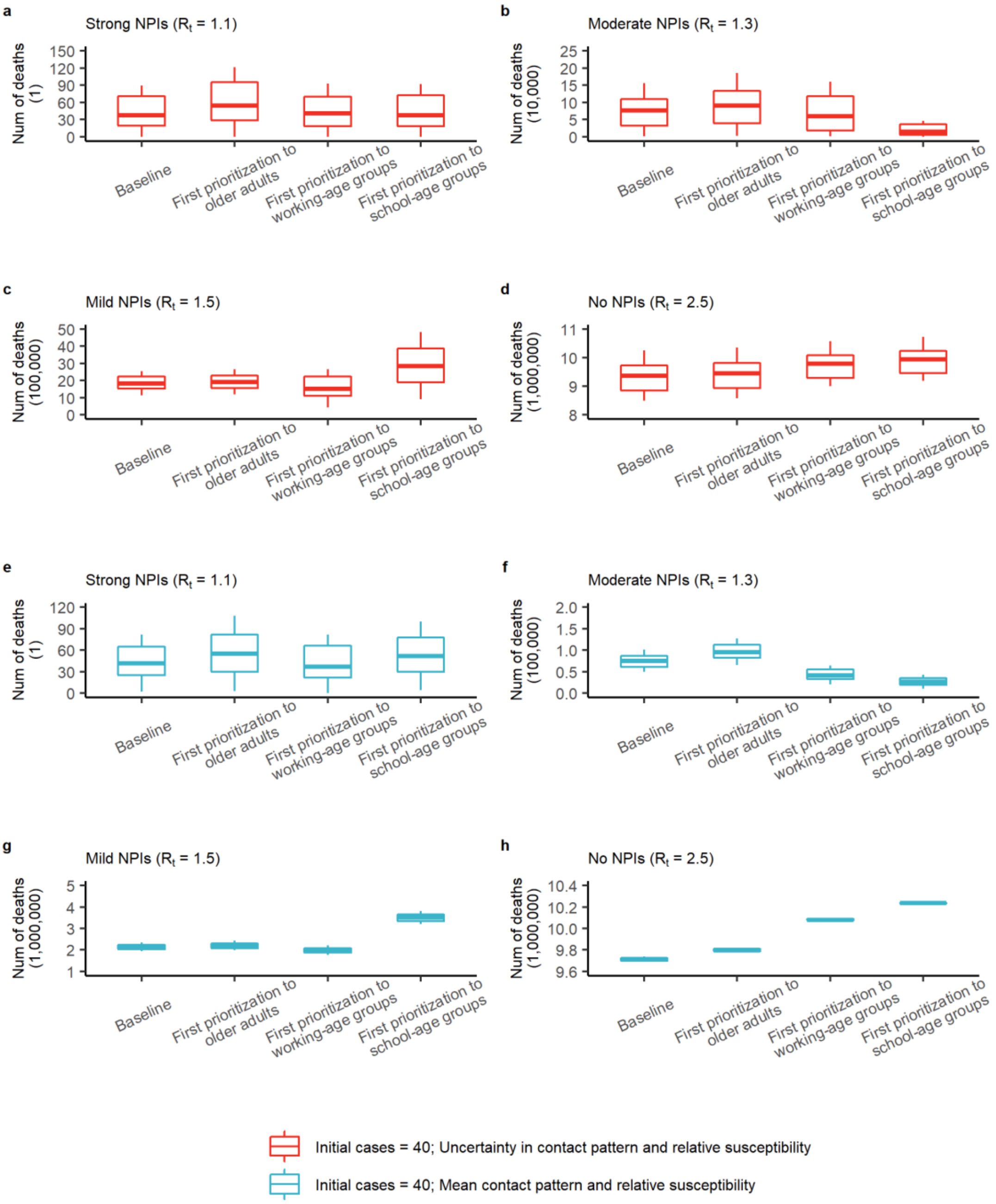

Supplementary Figure.4 Overall impact of vaccination prioritizations on cumulative COVID-19 deaths provided 6 million doses administered/day and 40 initial cases.

The baseline scenario corresponds to first prioritizing older adults and individuals with underlying conditions. a)-d) The number of deaths for scenarios with initial R_t=1.1, 1.3, 1.5 and 2.5, respectively. The orange boxplots in these panels are obtained by running 200 stochastic simulations of the model, where in each simulation the susceptibility to infection by age is sampled from its posterior distribution estimated in Ref. [4][4], and the contact matrix is sampled from the bootstrapped contact matrices presented in Ref. [1]. e)-i) The number of deaths for

scenarios with initial R_t=1.1, 1.3, 1.5 and 2.5, respectively. The green boxplots in these panels are obtained by running 200 stochastic simulations of the model, where in each simulation the susceptibility to infection by age is equal to the mean value estimated in Ref. [4], and the contact matrix is equal to the mean contact matrix presented in Ref. [1]. This allows us to assess the impact of the uncertainty on the estimates of the susceptibility to infection by age and of the age-mixing patterns on our results.

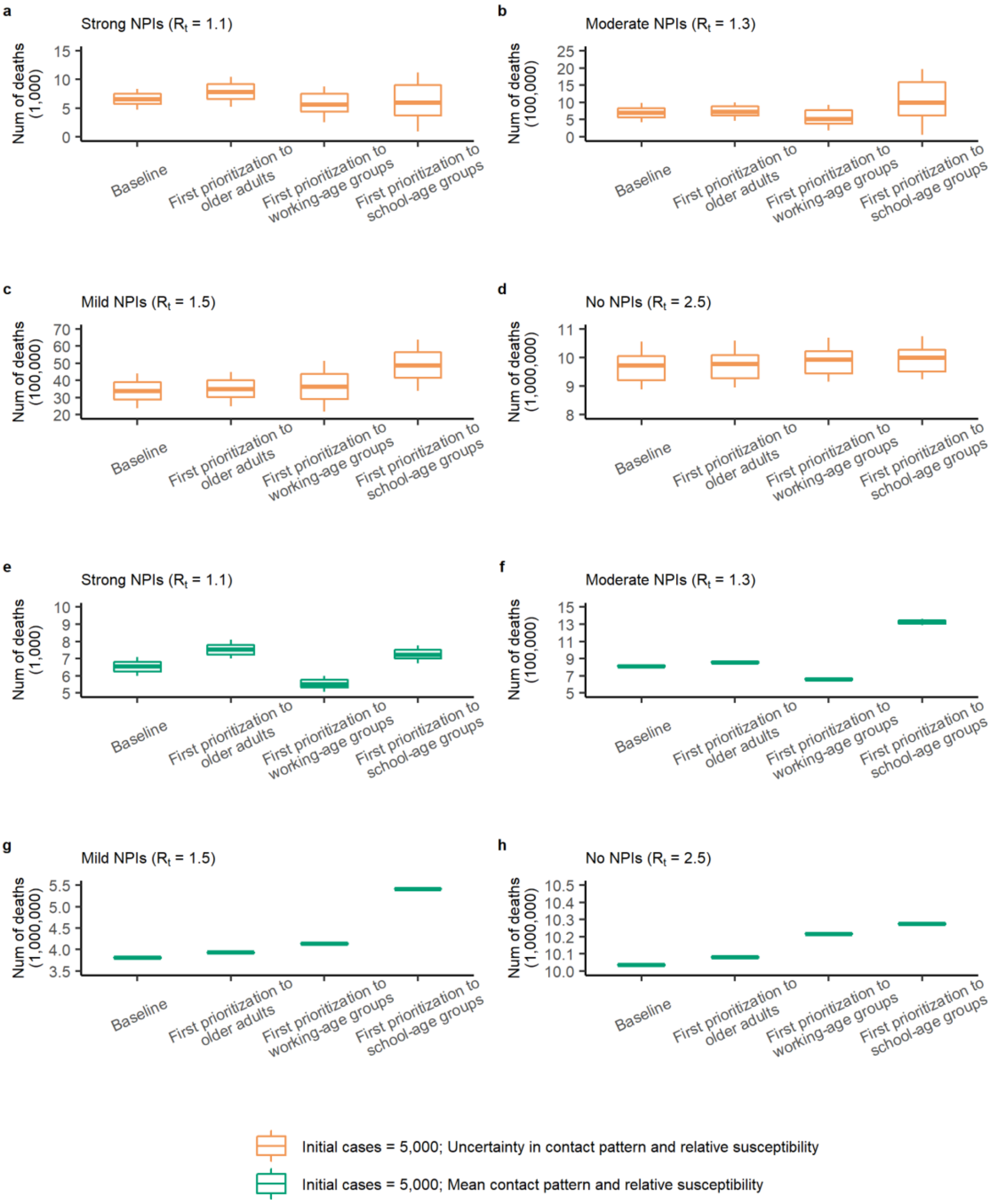

Supplementary Figure.5 Overall impact of vaccination prioritizations on cumulative COVID-19 deaths provided 6 million doses administered/day and 5,000 initial cases.

The baseline scenario corresponds to first prioritizing older adults and individuals with underlying conditions. a)-d) The number of deaths for scenarios with initial R_t=1.1, 1.3, 1.5 and 2.5, respectively. The orange boxplots in these panels are obtained by running 200 stochastic simulations of the model, where in each simulation the susceptibility to infection by age is sampled from its posterior distribution estimated in Ref. [4], and the contact matrix is sampled from the bootstrapped contact matrices presented in Ref. [1]. e)-i) The number of deaths for

scenarios with initial R_t=1.1, 1.3, 1.5 and 2.5, respectively. The green boxplots in these panels are obtained by running 200 stochastic simulations of the model, where in each simulation the susceptibility to infection by age is equal to the mean value estimated in Ref. [4], and the contact matrix is equal to the mean contact matrix presented in Ref. [1]. This allows us to assess the impact of the uncertainty on the estimates of the susceptibility to infection by age and of the age-mixing patterns on our results.

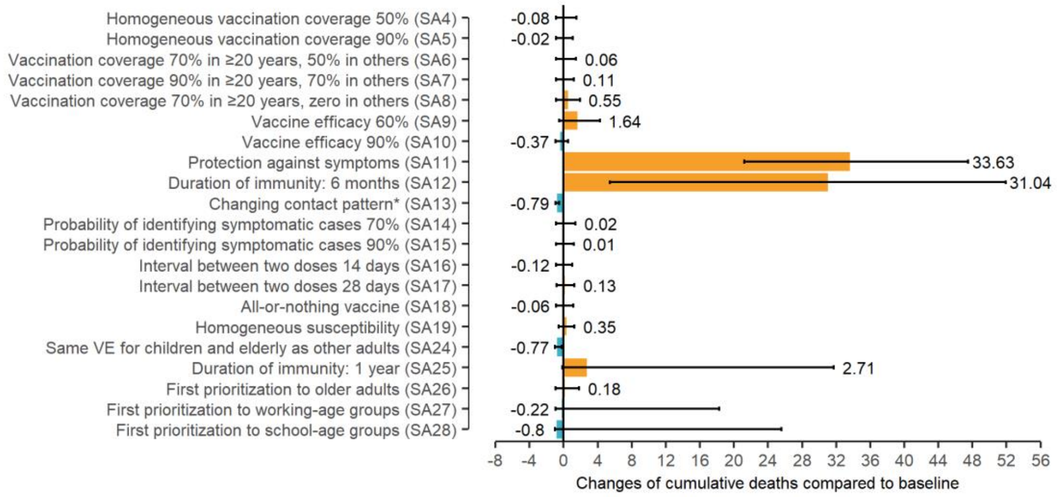

Supplementary Figure.6 Changes of the cumulative number of COVID-19 deaths estimated in the different sensitivity analyses, compared to the main analysis in the presence of moderate NPIs (R_t=1.3).

Number denotes median, and error bars denote quantiles 0.025 and 0.975. *In our main analysis, we us age-mixing patterns specific to China quantified during the pre-pandemic period (presented in Ref. [1]). Should a new COVID-19 wave start to unfold in China, it is unclear to what extent pre-pandemic contact patterns could be representative of such a situation. Therefore, we have added a sensitivity analysis where we assume the mixing patterns estimated in Shanghai in March 2020 (presented in Ref. [6]), when schools were still closed as a response to the COVID-19 pandemic.

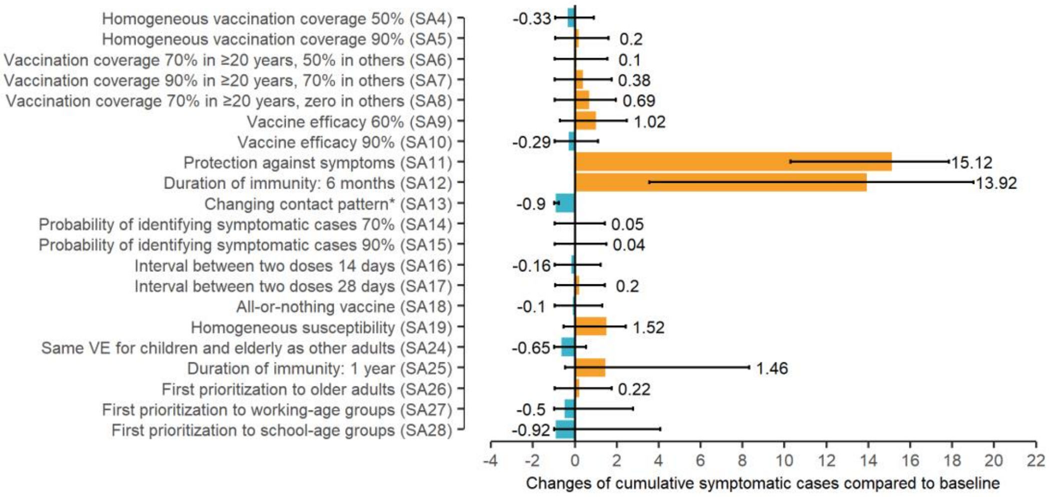

Supplementary Figure.7 Changes of the cumulative number of COVID-19 symptomatic cases estimated in the different sensitivity analyses and in the main analysis in the presence of moderate NPIs (R_t=1.3)

SE: sensitivity analysis. Number denotes median, and error bars denote quantiles 0.025 and 0.975. *In our main analysis, we us age-mixing patterns specific to China quantified during the pre-pandemic period (presented in Ref. [1]). Should a new COVID-19 wave start to unfold in China, it is unclear to what extent pre-pandemic contact patterns could be representative of such a situation. Therefore, we have added a sensitivity analysis where we assume the mixing patterns estimated in Shanghai in March 2020 (presented in Ref. [6]), when schools were still closed as a response to the COVID-19 pandemic.

Supplementary file 7. COVID-19 vaccine doses distributed over time in China

The Joint Prevention and Control Mechanism of the State Council in China released a three-step strategy for COVID-19 vaccine rollout [24]. We systematically collected information about COVID-19 vaccination in China from the website of the State Council and National Health Commission of the People's Republic of China [24,25]. The number of daily doses administered is less than 3 million in the first stage and shows a growing trend from 3 to 10 million, with a daily average of about 6 million doses over the period between late March and middle May, 2021. As of June 1, a total of 681.9 million doses have been administered.

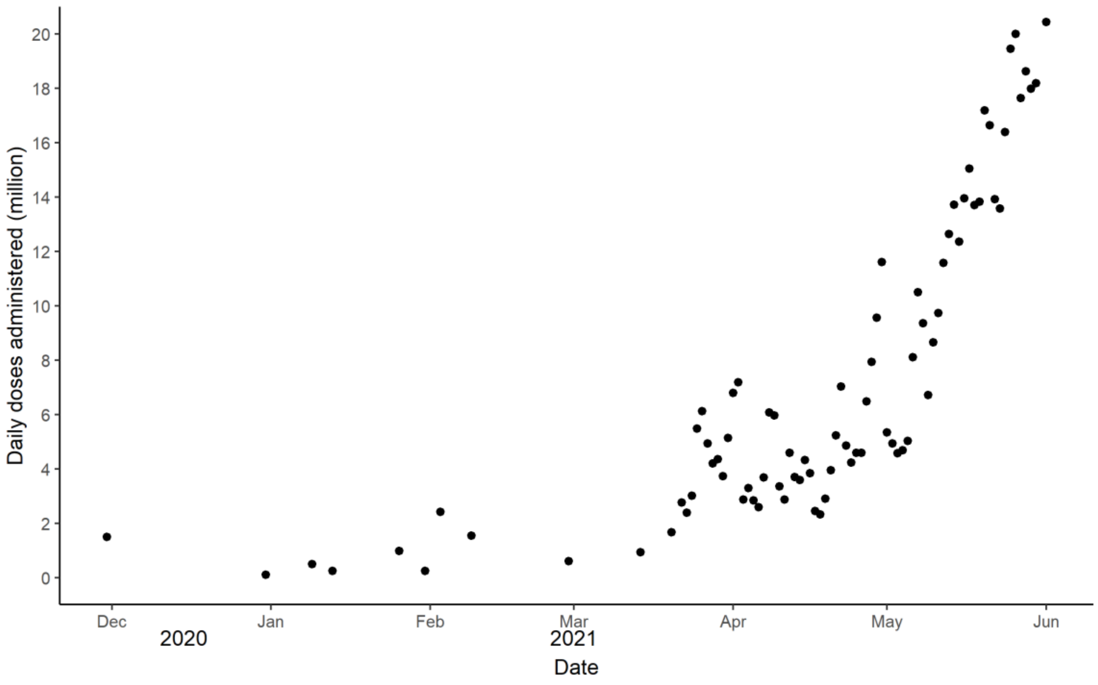

Supplementary Figure. 8 Doses of COVID-19 vaccines administered per day in China, as of June 1, 2021

References

1. Zhang, J. et al. Patterns of human social contact and contact with animals in Shanghai, China. *Sci Rep* **9**, 15141 (2019).
2. World Population Prospects 2019. *Population Division, Department of Economic and Social Affairs, United Nations* https://population.un.org/wpp/ (2019).
3. Yang, J. et al. Who should be prioritized for COVID-19 vaccination in China? A descriptive study. *BMC Med* **19**, 45 (2021).
4. Hu, S. et al. Infectivity, susceptibility, and risk factors associated with SARS-CoV-2 transmission under intensive contact tracing in Hunan, China. *Nature Communications* **12**, 1533 (2021).
5. Sun, K. et al. Transmission heterogeneities, kinetics, and controllability of SARS-CoV-2. *Science* **371**, eabe2424 (2021).
6. Zhang, J. et al. The impact of relaxing interventions on human contact patterns and SARS-CoV-2 transmission in China. *Science Advances* **7**, eabe2584 (2020).
7. Wu, J.T., Leung, K. & Leung, G.M. Nowcasting and forecasting the potential domestic and international spread of the 2019-nCoV outbreak originating in Wuhan, China: a modelling study. *Lancet* **395**, 689-697 (2020).
8. Li, Q. et al. Early Transmission Dynamics in Wuhan, China, of Novel Coronavirus–Infected Pneumonia. *N Engl J Med* **382**, 1199-1207 (2020).
9. Abbott, S., Hellewell, J., Munday, J., group, C.n.w. & Funk, S. The transmissibility of novel Coronavirus in the early stages of the 2019-20 outbreak in Wuhan: Exploring initial point-source exposure sizes and durations using scenario analysis. *Wellcome Open Res* **5**, 17 (2020).
10. Chinazzi, M. et al. The effect of travel restrictions on the spread of the 2019 novel coronavirus (COVID-19) outbreak. *Science* **368**, 395-400 (2020).
11. Natsuko, I. et al. Report 3: Transmissibility of 2019-nCoV. Imperial College London. https://doi.org/10.25561/77148 (2020)
12. World Health Organization. Report of the WHO-China Joint Mission on Coronavirus Disease 2019 (COVID-19). https://www.who.int/publications/i/item/report-of-the-who-china-joint-mission-on-coronavirus-disease-2019-(covid-19) (2021).
13. Xia, S. et al. Safety and immunogenicity of an inactivated SARS-CoV-2 vaccine, BBIBP-CorV: a randomised, double-blind, placebo-controlled, phase 1/2 trial. *Lancet Infect Dis* **21**, 39-51 (2021).
14. Sinopharm COVID-19 vaccine licensed in China. *Sinopharm* http://www.sinopharm.com/s/1223-3763-38840.html (2021).
15. Yang, P. et al. Influenza vaccine effectiveness against medically-attended influenza illness during the 2012-2013 season in Beijing, China. *Vaccine* **32**, 5285-5289 (2014).
16. The Central People's Government of the People's Republic of China. Report of H1N1 pandemic influenza vaccination from Ministry of Health. http://www.gov.cn/gzdt (2020).
17. Poletti, P. et al. Association of Age With Likelihood of Developing Symptoms and Critical

Disease Among Close Contacts Exposed to Patients With Confirmed SARS-CoV-2 Infection in Italy. *JAMA Netw Open* **4**, e211085 (2021).

18. Yang, J. et al. Disease burden and clinical severity of the first pandemic wave of COVID-19 in Wuhan, China. *Nat Commun* **11**, 5411 (2020).
19. Guan, W. et al. Clinical Characteristics of Coronavirus Disease 2019 in China. *N Engl J Med* **382**, 1708-1720 (2020).
20. Diekmann, O., Heesterbeek, J.A. & Metz, J.A. On the definition and the computation of the basic reproduction ratio R0 in models for infectious diseases in heterogeneous populations. *Journal of Mathematical Biology* **28**, 365-382 (1990).
21. Trentini, F. et al. Healthcare strain and intensive care during the COVID-19 outbreak in the Lombardy region: a retrospective observational study on 43,538 hospitalized patients. Preprint at *medRxiv* http://medrxiv.org/content/early/2020/11/07/2020.11.06.20149690.abstract (2020).
22. Poletti, P. et al. Age-specific SARS-CoV-2 infection fatality ratio and associated risk factors, Italy, February to April 2020. *Euro Surveill* **25**, 2001383 (2020).
23. Zhang, J. et al. Evolving epidemiology and transmission dynamics of coronavirus disease 2019 outside Hubei province, China: a descriptive and modelling study. *Lancet Infect. Dis.* **7**, 793-802 (2020).
24. Press Conference of the Joint Prevention and Control Mechanism of the State Council. http://www.gov.cn/xinwen/gwylflkjz144/index.htm (2021).
25. National Health Commission of the People's Republic of China. COVID-19 vaccination reports. http://www.nhc.gov.cn/xcs/yqjzqk/list_gzbd.shtml (2021).
