# Supplementary material for: Despite vaccination, China needs non-pharmaceutical interventions to prevent widespread outbreaks of COVID-19 in 2021
Source: Nat Hum Behav. 2021 Jun 22;5(8):1009–20. doi: 10.1038/s41562-021-01155-z (PMC8373613; doi:10.1038/s41562-021-01155-z)
Supplement: Supplementary file 3 — Peer review information [file 41562_2021_1155_MOESM3_ESM.pdf]

naturerearch

Peer Review Information

Journal: Nature Human Behaviour
Manuscript Title: Despite vaccination, China will have to maintain non-pharmaceutical interventions to prevent widespread outbreaks of COVID-19 in 2021
Corresponding author name(s): Marco Ajelli & Hongjie Yu

Editorial Notes:

Transferred manuscripts	This manuscript has been previously reviewed at another journal that is not operating a transparent peer review scheme. This document only contains reviewer comments, rebuttal and decision letters for versions considered at Nature Human Behaviour.

Reviewer Comments & Decisions:

Decision Letter, initial version:

Our ref: NATHUMBEHAV-210515300-T

19th May 2021

Dear Professor Yu,

Thank you once again for transferring your revised manuscript "To what extent do we need to rely on non-pharmaceutical interventions while COVID-19 vaccines roll out in 2021?" (NATHUMBEHAV-210515300-T) to Nature Human Behaviour. It has now been seen by one of the original referees, and their comments are below. As you can see, the reviewer finds that the paper has improved in revision. We will therefore be happy in principle to publish it in Nature Human Behaviour, pending minor revisions to comply with our editorial and formatting guidelines

We are now performing detailed checks on your paper and will send you a checklist detailing our editorial and formatting requirements in the next two days. Please do not upload the final materials and make any revisions until you receive this additional information from us.

Please do not hesitate to contact me if you have any questions.

Sincerely,

Arunas Radzvilavicius

Editor
Nature Human Behaviour

Reviewer #1 (Remarks to the Author):

After the revision, the authors have added a lot of important content (e.g., sensitivity analyses considering three different scenarios of vaccine coverage and a 28-day interval between the first and second vaccine dose, and clarification of the study limitations) of the manuscript and provided very detailed answers to the comments raised by the two reviewers. Therefore, the manuscript has been substantially improved. I suggest consideration to publish into Nature Human Behaviour. There is only one issue: maybe the authors could improve the figure quality.

Decision letter, final requests:

** Please ensure you delete the link to your author homepage in this e-mail if you wish to forward it to your co-authors. **

Our ref: NATHUMBEHAV-210515300-T

21st May 2021

Dear Dr. Yu,

Thank you for your patience as we've prepared the guidelines for final submission of your Nature Human Behaviour manuscript, "To what extent do we need to rely on non-pharmaceutical interventions while COVID-19 vaccines roll out in 2021?" (NATHUMBEHAV-210515300-T). Please carefully follow the step-by-step instructions provided in the attached file, and add a response in each row of the table to indicate the changes that you have made. Please also check and comment on any additional marked-up edits we have proposed within the text. Ensuring that each point is addressed will help to ensure that your revised manuscript can be swiftly handed over to our production team.

We would like to start working on your revised paper, with all of the requested files and forms, as soon as possible (preferably within two weeks). Please get in contact with us if you anticipate delays.

When you upload your final materials, please include a point-by-point response to any remaining reviewer comments.

If you have not done so already, please alert us to any related manuscripts from your group that are under consideration or in press at other journals, or are being written up for submission to other journals (see: https://www.nature.com/nature-research/editorial-policies/plagiarism#policy-on-duplicate-publication for details).

Nature Human Behaviour offers a Transparent Peer Review option for new original research manuscripts submitted after December 1st, 2019. As part of this initiative, we encourage our authors to support increased transparency into the peer review process by agreeing to have the reviewer

comments, author rebuttal letters, and editorial decision letters published as a Supplementary item. When you submit your final files please clearly state in your cover letter whether or not you would like to participate in this initiative. Please note that failure to state your preference will result in delays in accepting your manuscript for publication.

In recognition of the time and expertise our reviewers provide to Nature Human Behaviour's editorial process, we would like to formally acknowledge their contribution to the external peer review of your manuscript entitled "To what extent do we need to rely on non-pharmaceutical interventions while COVID-19 vaccines roll out in 2021?". For those reviewers who give their assent, we will be publishing their names alongside the published article.

Cover suggestions

As you prepare your final files we encourage you to consider whether you have any images or illustrations that may be appropriate for use on the cover of Nature Human Behaviour.

Covers should be both aesthetically appealing and scientifically relevant, and should be supplied at the best quality available. Due to the prominence of these images, we do not generally select images featuring faces, children, text, graphs, schematic drawings, or collages on our covers.

We accept TIFF, JPEG, PNG or PSD file formats (a layered PSD file would be ideal), and the image should be at least 300ppi resolution (preferably 600-1200 ppi), in CMYK colour mode.

If your image is selected, we may also use it on the journal website as a banner image, and may need to make artistic alterations to fit our journal style.

Please submit your suggestions, clearly labeled, along with your final files. We'll be in touch if more information is needed.

ORCID

Non-corresponding authors do not have to link their ORCIDs but are encouraged to do so. Please note that it will not be possible to add/modify ORCIDs at proof. Thus, please let your co-authors know that if they wish to have their ORCID added to the paper they must follow the procedure described in the following link prior to acceptance: https://www.springernature.com/gp/researchers/orcid/orcid-for-nature-research

Nature Human Behaviour has now transitioned to a unified Rights Collection system which will allow our Author Services team to quickly and easily collect the rights and permissions required to publish your work. Approximately 10 days after your paper is formally accepted, you will receive an email in providing you with a link to complete the grant of rights. If your paper is eligible for Open Access, our Author Services team will also be in touch regarding any additional information that may be required to arrange payment for your article. Please note that you will not receive your proofs until the publishing agreement has been received through our system.

Please note that <i>Nature Human Behaviour</i> is a Transformative Journal (TJ). Authors may publish their research with us through the traditional subscription access route or make their paper

immediately open access through payment of an article-processing charge (APC). Authors will not be required to make a final decision about access to their article until it has been accepted. Find out more about Transformative Journals

<B>Authors may need to take specific actions to achieve compliance with funder and institutional open access mandates. For submissions from January 2021, if your research is supported by a funder that requires immediate open access (e.g. according to Plan S principles) then you should select the gold OA route, and we will direct you to the compliant route where possible. For authors selecting the subscription publication route our standard licensing terms will need to be accepted, including our self-archiving policies. Those standard licensing terms will supersede any other terms that the author or any third party may assert apply to any version of the manuscript.

For information regarding our different publishing models please see our Transformative Journals page. If you have any questions about costs, Open Access requirements, or our legal forms, please contact ASJournals@springernature.com.

Please use the following link for uploading these materials:
[REDACTED]

If you have any further questions, please feel free to contact me.

Best regards,
Chloe Knight
Editorial Assistant
Nature Human Behaviour

On behalf of

Arunas Radzvilavicius
Editor
Nature Human Behaviour

Reviewer #1:
Remarks to the Author:
After the revision, the authors have added a lot of important content (e.g., sensitivity analyses considering three different scenarios of vaccine coverage and a 28-day interval between the first and second vaccine dose, and clarification of the study limitations) of the manuscript and provided very detailed answers to the comments raised by the two reviewers. Therefore, the manuscript has been

substantially improved. I suggest consideration to publish into Nature Human Behaviour. There is only one issue: maybe the authors could improve the figure quality.

Final Decision Letter:

Dear Professor Yu,

We are pleased to inform you that your Article "Despite vaccination, China will have to maintain non-pharmaceutical interventions to prevent widespread outbreaks of COVID-19 in 2021", has now been accepted for publication in Nature Human Behaviour.

Before your manuscript is typeset, we will edit the text to ensure it is intelligible to our wide readership and conforms to house style. We look particularly carefully at the titles of all papers to ensure that they are relatively brief and understandable.

Once your manuscript is typeset and you have completed the appropriate grant of rights, you will receive a link to your electronic proof via email with a request to make any corrections within 48 hours. If, when you receive your proof, you cannot meet this deadline, please inform us at rjsproduction@springernature.com immediately. Once your paper has been scheduled for online publication, the Nature press office will be in touch to confirm the details.

Acceptance of your manuscript is conditional on all authors' agreement with our publication policies (see http://www.nature.com/nathumbehav/info/gta). In particular your manuscript must not be published elsewhere and there must be no announcement of the work to any media outlet until the publication date (the day on which it is uploaded onto our web site).

Please note that <i>Nature Human Behaviour</i> is a Transformative Journal (TJ). Authors may publish their research with us through the traditional subscription access route or make their paper immediately open access through payment of an article-processing charge (APC). Authors will not be required to make a final decision about access to their article until it has been accepted. Find out more about Transformative Journals

<B>Authors may need to take specific actions to achieve compliance with funder and institutional open access mandates. For submissions from January 2021, if your research is supported by a funder that requires immediate open access (e.g. according to Plan S principles) then you should select the gold OA route, and we will direct you to the compliant route where possible. For authors selecting the subscription publication route our standard licensing terms will need to be accepted, including our self-archiving policies. Those standard licensing terms will supersede any other terms that the author or any third party may assert apply to any version of the manuscript.

If you have posted a preprint on any preprint server, please ensure that the preprint details are

updated with a publication reference, including the DOI and a URL to the published version of the article on the journal website.

An online order form for reprints of your paper is available at https://www.nature.com/reprints/author-reprints.html. All co-authors, authors' institutions and authors' funding agencies can order reprints using the form appropriate to their geographical region.

We welcome the submission of potential cover material (including a short caption of around 40 words) related to your manuscript; suggestions should be sent to Nature Human Behaviour as electronic files (the image should be 300 dpi at 210 x 297 mm in either TIFF or JPEG format). Please note that such pictures should be selected more for their aesthetic appeal than for their scientific content, and that colour images work better than black and white or grayscale images. Please do not try to design a cover with the Nature Human Behaviour logo etc., and please do not submit composites of images related to your work. I am sure you will understand that we cannot make any promise as to whether any of your suggestions might be selected for the cover of the journal.

You can now use a single sign-on for all your accounts, view the status of all your manuscript submissions and reviews, access usage statistics for your published articles and download a record of your refereeing activity for the Nature journals.

To assist our authors in disseminating their research to the broader community, our SharedIt initiative provides you with a unique shareable link that will allow anyone (with or without a subscription) to read the published article. Recipients of the link with a subscription will also be able to download and print the PDF.

As soon as your article is published, you will receive an automated email with your shareable link.

In approximately 10 business days you will receive an email with a link to choose the appropriate publishing options for your paper and our Author Services team will be in touch regarding any additional information that may be required.

You will not receive your proofs until the publishing agreement has been received through our system.

If you have any questions about our publishing options, costs, Open Access requirements, or our legal forms, please contact ASJournals@springernature.com

We look forward to publishing your paper.

With best regards,

Arunas Radzvilavicius
Editor
Nature Human Behaviour

P.S. Click on the following link if you would like to recommend Nature Human Behaviour to your librarian http://www.nature.com/subscriptions/recommend.html#forms

** Visit the Springer Nature Editorial and Publishing website at www.springernature.com/editorial-and-publishing-jobs for more information about our career opportunities. If you have any questions please click here.**
